# Metabolic syndrome in relation to dietary acid load: a dose–response meta-analysis of observational studies

**DOI:** 10.3389/fnut.2023.1233746

**Published:** 2023-08-11

**Authors:** Sulieman Ibraheem Shelash Al-Hawary, Faris Mushabab, Shahabe Saquib Abullais, Raed H. Althomali, Ebraheem Abdu Musad Saleh, Serar Nassir Alnajjar, Khulood H. Oudaha, Rosario Mireya Romero-Parra, Beneen M. Hussien, Nazila Garousi

**Affiliations:** ^1^Department of Business Administration, Business School, Al al-Bayt University, Mafraq, Jordan; ^2^Department of Periodontics, Albaha University, Al Bahah, Saudi Arabia; ^3^Department of Periodontics and Community Dental Sciences, College of Dentistry, King Khalid University, Abha, Saudi Arabia; ^4^Department of Chemistry, College of Arts and Science, Prince Sattam Bin Abdulaziz University, Wadi Al-Dawasir, Saudi Arabia; ^5^College of Dentistry, Al-Bayan University, Baghdad, Iraq; ^6^Pharmaceutical Chemistry Department, College of Pharmacy, Al-Ayen University, Nasiriyah, Iraq; ^7^Department of General Studies, Universidad Continental, Lima, Peru; ^8^Medical Laboratory Technology Department, College of Medical Technology, The Islamic University, Najaf, Iraq; ^9^Department of Clinical Nutrition, School of Nutrition and Food Science, Isfahan University of Medical Sciences, Isfahan, Iran

**Keywords:** dietary acid load, NEAP, PRAL, metabolic syndrome, meta-analysis

## Abstract

**Background and aim:**

Several studies have identified that dietary acid load (DAL) may be associated with the odds of metabolic syndrome (MetS); however, the evidence is inconclusive. This dose–response meta-analysis aimed to examine the relation of DAL to MetS.

**Methods:**

A systematic literature search was carried out in PubMed and Scopus up to April 2023 for pertinent studies evaluating the relation of DAL scores, including potential renal acid load (PRAL) and net endogenous acid production (NEAP), to the odds of MetS. The odds ratios (OR) with 95% confidence intervals (CI) were pooled using a random-effects meta-analysis to test the association.

**Results:**

Eight studies, with an overall sample size of 31,351 participants, were included in this meta-analysis. Higher DAL scores were significantly related to the elevated odds of MetS (NEAP: OR = 1.42, 95%CI = 1.12–1.79; PRAL: OR = 1.76, 95%CI = 1.11–2.78), with significant evidence of heterogeneity across studies. The linear dose–response analysis proposed that a 10 mEq/day elevation in NEAP and PRAL was linked to a 2% (OR = 1.02, 95%CI = 1.001–1.05) and 28% (OR = 1.28, 95%CI = 1.11–1.47) increased odds of MetS, respectively. No non-linear association was observed between MetS and NEAP (P-non-linearity = 0.75) and PRAL (P-non-linearity = 0.92).

**Conclusion:**

This study revealed a significant direct relationship between DAL and MetS. Therefore, lower acidogenic diets are suggested for the prevention of MetS.

## 1. Introduction

Metabolic syndrome (MetS), characterized by a cluster of metabolic abnormalities including insulin resistance, obesity, dyslipidemia, and hypertension, has become a prominent concern worldwide, owing to its prevalence and association with chronic diseases such as cardiovascular diseases, type 2 diabetes, and stroke (1–3). Genetics and environmental factors are both involved in the etiology of this disorder (4). Given its high prevalence and adverse health consequences, it is essential to identify preventive approaches against MetS.

Evidence has suggested the contribution of numerous dietary factors to the development of MetS; however, the focus should be on the dietary patterns rather than on single food items/ingredients as dietary patterns yield the whole effect of diet by considering the complex interactions between various food components (5–7). In recent years, the role of dietary acid load (DAL), as measured by the net endogenous acid production (NEAP) and potential renal acid load (PRAL), has been of particular interest. NEAP and PRAL reflect the dietary content of acid-or base-forming compounds that may affect the acid–base balance in the body (8). The PRAL is computed based on the dietary consumption of magnesium, potassium, phosphorous, calcium, and protein, while NEAP is computed using the intakes of potassium and total protein, which partly play a role in metabolic acidosis (9). A diet high in animal protein and grains and low in fruits and vegetables typically leads to a high acid load (8). Diet has been demonstrated to be a leading contributor to variations in endogenous acid production in different people (10).

Although associations between DAL and individual features of MetS have been reported, evidence regarding the link between DAL and the overall odds of MetS is scarce and highly controversial (11–13). The relation of DAL to MetS may differ by race, gender, geographic region, and other demographic features of various populations. Therefore, this meta-analysis aims to summarize the current evidence on the association between DAL, defined by NEAP and PRAL, and the odds of MetS.

## 2. Materials and methods

This meta-analysis was implemented by following the Preferred Reporting Items for Systematic Reviews and Meta-Analyzes (PRISMA) protocols (14).

### 2.1. Search strategy

We performed a systematic literature search with no language restriction through the Scopus and PubMed databases to find all pertinent studies published up to April 2023. The search strategy was as follows: ((((((((((((“Dietary acid load”[Title/Abstract]) OR (“dietary acid–base load”[Title/Abstract])) OR (“dietary acidity”[Title/Abstract])) OR (“net acid load”[Title/Abstract])) OR (“acid excretion”[Title/Abstract])) OR (“potential renal acid load”[Title/Abstract])) OR (PRAL [Title/Abstract])) OR (“net endogenous acid production”[Title/Abstract])) OR (NEAP [Title/Abstract])) OR (“protein to potassium ratio”[Title/Abstract])) OR (“protein/ potassium ratio”[Title/Abstract])) OR (“potential renal acid load”[Title/Abstract])) AND (((“Metabolic Syndrome”[Mesh]) OR (metabolic syndrome [Title/Abstract])) OR (insulin resistance syndrome [Title/Abstract])). The reference lists of the associated publications were also screened manually to evade missing any study.

### 2.2. Inclusion criteria

For the present meta-analysis, publications were expected to meet all the following criteria to be eligible for inclusion: (1) observational studies (prospective, case–control, or cross-sectional) investigating the relation of DAL, assessed by NEAP or PRAL (15, 16), to MetS; (2), studied reported odds ratios (OR), relative risk (RR), or hazard ratios (HR) and their 95% confidence intervals (CI) (or provided sufficient data to calculate them) for the relations of DAL indices, NEAP or PRAL, to the odds of MetS. We excluded reviews, letters, comments, conference papers, animal studies, and studies with irrelevant exposure/outcome during the screening of studies.

### 2.3. Data extraction and quality assessment

Data were extracted by two independent reviewers with the use of a standardized data extraction form, and inconsistencies were resolved by discussion among all authors. The following information was obtained from each publication: first author, publication year, type of exposure (NEAP or PRAL), mean or range of age, total sample size, number of MetS cases, gender, type of study, country, method of dietary assessment, the definition used for the diagnosis of MetS, confounder variables adjusted for in analyzes, and effect sizes (OR, RR, or HR with their 95%CI). If a publication applied both NEAP and PRAL for the evaluation of DAL, both effect sizes for NEAP and PRAL were extracted separately. When necessary, we contacted the corresponding authors to obtain publications. The quality of the studies was evaluated with the use of the Newcastle–Ottawa scale (NOS), in which scores of 0–3, 4–6, and 7–9 were considered as low, moderate, and high quality, respectively (17).

### 2.4. Statistical analysis

The included studies reported effect sizes for the associations in various models; for the present meta-analysis, we obtained the ORs and 95%CIs in the highest category of NEAP or PRAL scores, compared to the lowest category, in the most adjusted model. The ORs and 95%CIs in the highest vs. lowest N tiles of DAL were used as effect size in the meta-analysis to compute the pooled effect for the association. Heterogeneity across the studies was measured with the use of the Q-statistics and *I*^2^ values, and *I*^2^ > 50% or *p* < 0.1 were considered as statistically significant evidence of heterogeneity (18, 19). Because of the anticipated heterogeneity, data were pooled by the DerSimonian–Laird random-effects model (20). To instigate possible sources of heterogeneity, subgroup analysis by definition of MetS and the sex of participants was performed. Using the two-stage generalized least-squares trend estimation approach, a linear dose–response meta-analysis, as reported by Greenland and Longnecker (21), was carried out for the odds of MetS associated with each increment of 10 mEq/day in NEAP and PRAL. To obtain the overall average slope, study-specific slope lines were first computed, and these lines were then blended using a random-effects model (22). To investigate non-linear associations, restricted cubic splines for each study with ≥3 categories of exposure were computed with the use of three fixed knots at 10, 50, and 90% through the total distribution of reported exposure, then combined with the use of multivariate meta-analysis (23–25). The distribution of cases and non-cases, the mean or median of the NEAP or PRAL scores, and the ORs with the 95%CIs for at least three exposure categories were needed for the dose–response analysis. Publication bias was also evaluated by funnel plots and Egger’s test (26, 27). All statistical analyzes were performed with the Stata software (version 14). *p* < 0.05 was considered statistically significant for the relation of NEAP and PRAL to MetS.

## 3. Results

### 3.1. Characteristics of studies

**Figure 1:**
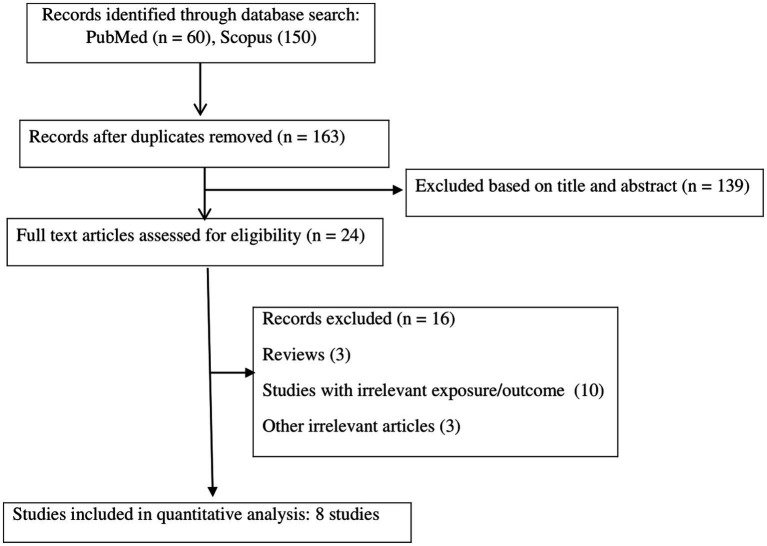
Flow chart for studies selection.

The systematic literature search yielded a total of 210 publications. Of these, 47 studies were duplicates, and 139 studies were irrelevant based on the titles/abstracts, and these were thus excluded. The full texts of 24 potentially pertinent publications were reviewed, and finally, a total of eight studies (8, 9, 11–13, 28–30), with 31,351 participants, published from 2015 to 2022, were included in the meta-analysis according to the inclusion criteria. The study by Arisawa et al. reported the results for men and women separately; thus, two effect sizes were extracted from this study (8). The flow diagram of the study selection is reported in Figure 1. All studies were cross-sectional in design. Among the studies, five were performed in Iran (9, 12, 28–30), two were performed in Japan (8, 11), and one was from Italy (13). All analyzed studies reported effect sizes that were controlled for the potential covariate except for the study by Sanz et al. (13), which was based on a crude analysis without adjustment for confounders. The effect sizes for NEAP and PRAL were available in seven studies (with eight effect sizes) (8, 9, 11, 13, 28–30) and seven studies (9, 11–13, 28–30), respectively. Two publications reported effect sizes only for women (9, 12), one only for men (28), four for the combination of both genders (11, 13, 29, 30), and one for men and women separately (8). Dietary assessment was based on a food frequency questionnaire (FFQ) in seven studies (8, 9, 12, 13, 28–30) and a diet history questionnaire in one study (11). The definition of MetS was based on the National Cholesterol Education Program Adult Treatment Panel III (NCEP ATP III) in five studies (9, 12, 13, 28, 29), Joint Interim Statement Criteria (JIS) of 2009 in two studies (8, 11), and International Diabetes Federation (IDF) in one study (30). The quality of the included studies was moderate to high, with NOS scores ranging from 7 to 9 (Supplementary Table S1). The characteristics of the studies are presented in Table 1.

**Table 1:** Characteristics of included studies.

Author	Year	Study design	Location	Sex	No. of participants	Cases	Age (mean ± sd or range)	Exposure type	Exposure assessment	MetS diagnosis	Adjustment
Arisawa et al.	2020	Cross-sectional	Japan	Men	14,042	3,155	35–69	NEAP	*FFQ*	Joint Interim Statement Criteria of 2009	Adjusted for age, study site, smoking and drinking habits, physical activity level, total energy intake, and school career, plus nutrient pattern (fiber, iron, potassium, and vitamins pattern) scores
				Women	14,105	1,472					
Sanz et al.	2022	Cross-sectional	Italy	Both	448	74	55–80	NEAP PRAL	*FFQ*	NCEP ATP III	Crude
Iwase et al.	2015	Cross-sectional	Japan	Both	149	67	65.7 ± 9.3	NEAP PRAL	Diet history questionnaire	Joint Interim Statement Criteria of 2009	Adjusted for age, sex, serum uric acid and creatinine, total energy intake, carbohydrate intake, and sodium intake
Jafari et al.	2021	Cross-sectional	Iran	Men	357	NR	>60 years	NEAP PRAL	*FFQ*	NCEP ATP III	Adjusted for age, smoking, physical activity, socioeconomic status, marital status, energy, disease, anti-diabetic drugs, thyroid drugs, and heart disease drugs
Mozaffari et al.	2019	Cross-sectional	Iran	Women	371	NR	20–50	NEAP PRAL	*FFQ*	NCEP ATP III	Adjusted for energy intake, age, marital status, socioeconomic status, and BMI
Rezazadegan et al.	2022	Cross-sectional	Iran	Both	203	79	12 to 18 y	NEAP PRAL	*FFQ*	IDF	Adjusted for age, sex, energy intake, physical activity, socioeconomic status, and BMI
Tangestani et al.	2022	Cross-sectional	Iran	Women	246	79	36.49 ± 8.38	PRAL	*FFQ*	NCEP ATP III	Adjusted for age, physical activity, and socio-economic, marital, and education status
Mohammadifard et al.	2020	Cross-sectional	Iran	Both	1,430	205	38.70 ± 10.66	NEAP PRAL	*FFQ*	NCEP ATP III	Adjusted for age, physical activity, and BMI

BMI, body mass index; FFQ, food frequency questionnaire; NEAP, net endogenous acid production; PRAL, potential renal acid load; NCEPATP III, National Cholesterol Education Program Adult Treatment Panel III; IDF, International Diabetes Federation.

### 3.2. Findings from the meta-analysis

**Figure 2:**
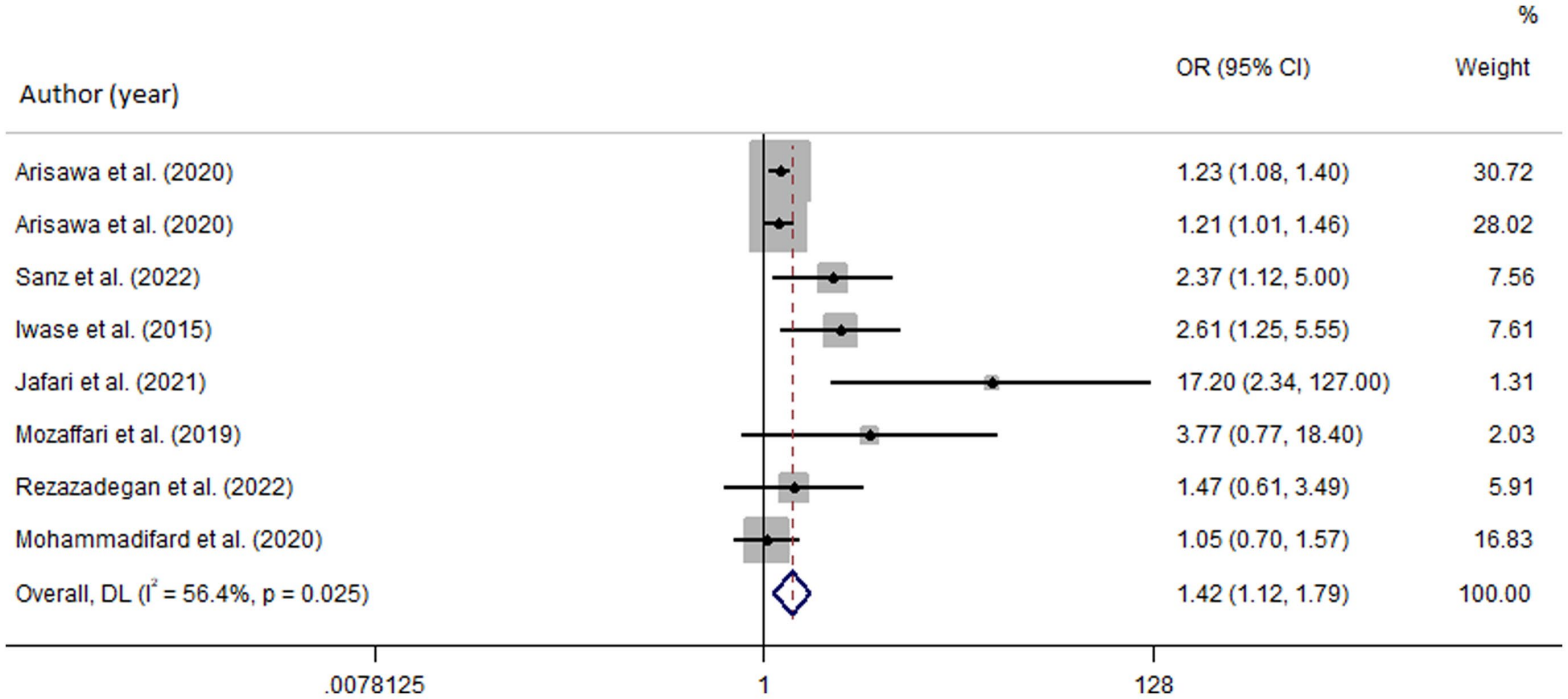
Forest plot of the pooled data for the association between high dietary acid load (based on NEAP) and odds of metabolic syndrome.

**Figure 3:**
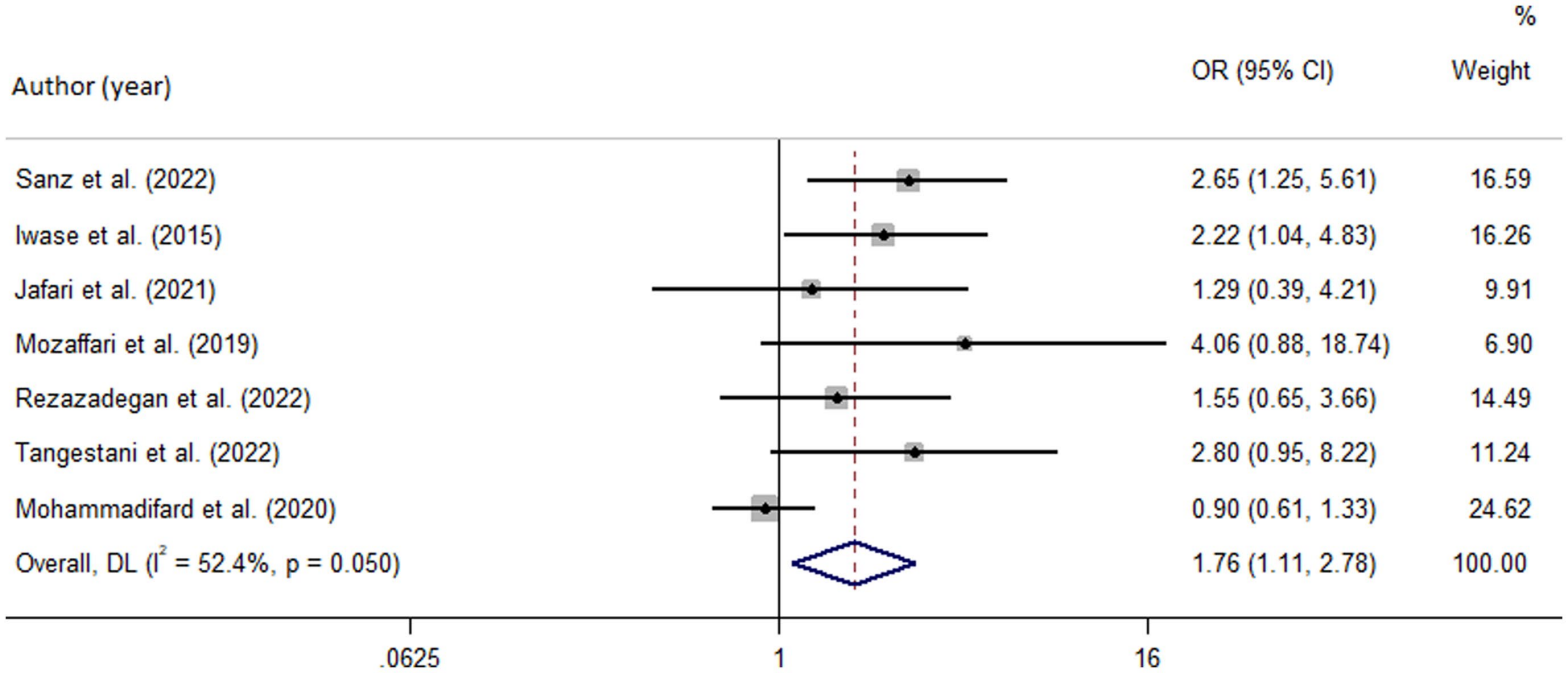
Forest plot of the pooled data for the association between high dietary acid load (based on PRAL) and odds of metabolic syndrome.

**Figure 4:**
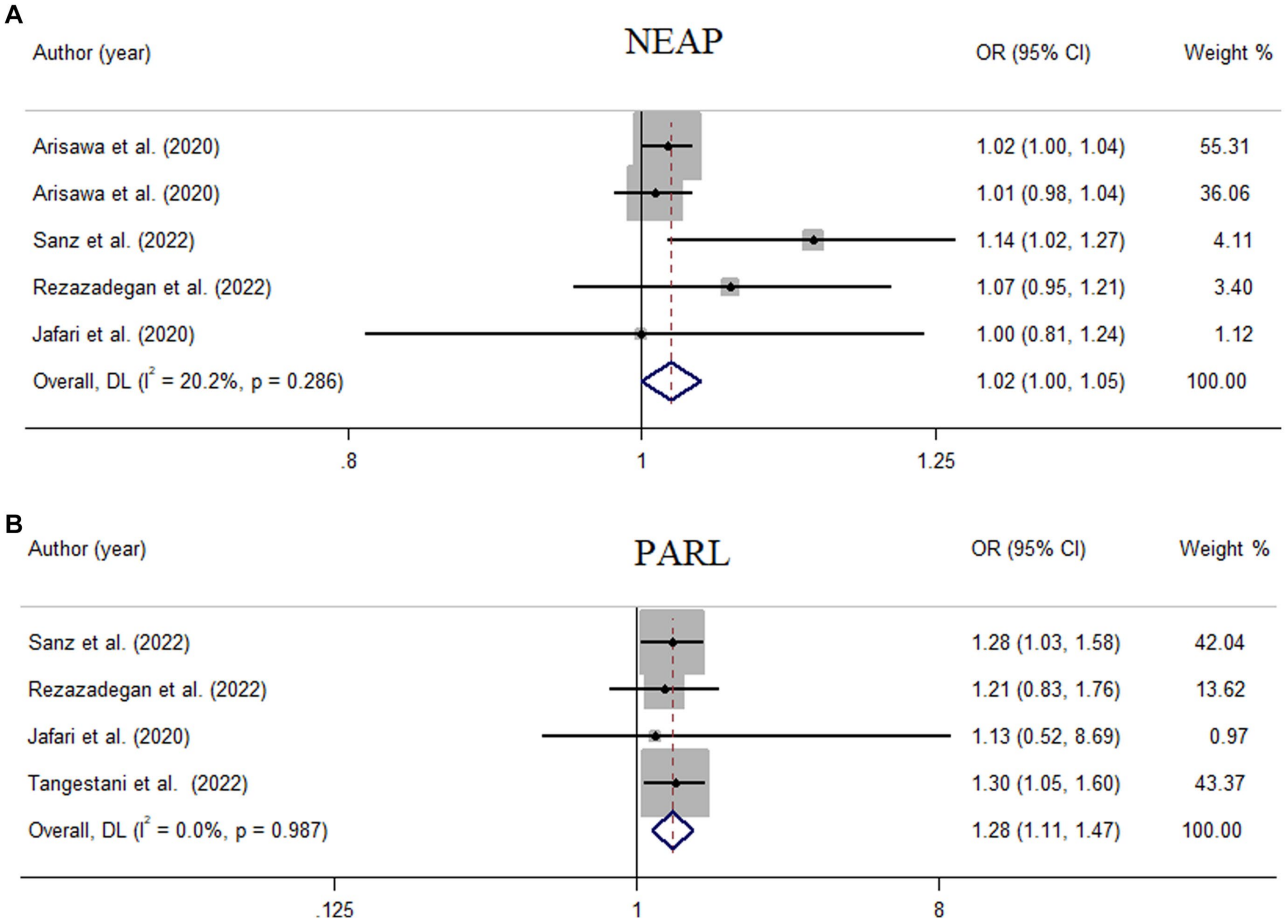
Meta-analysis of the linear association of dietary acid load for a 10-unit increment in NEAP **(A)** and PRAL **(B)** with odds of metabolic syndrome.

**Figure 5:**
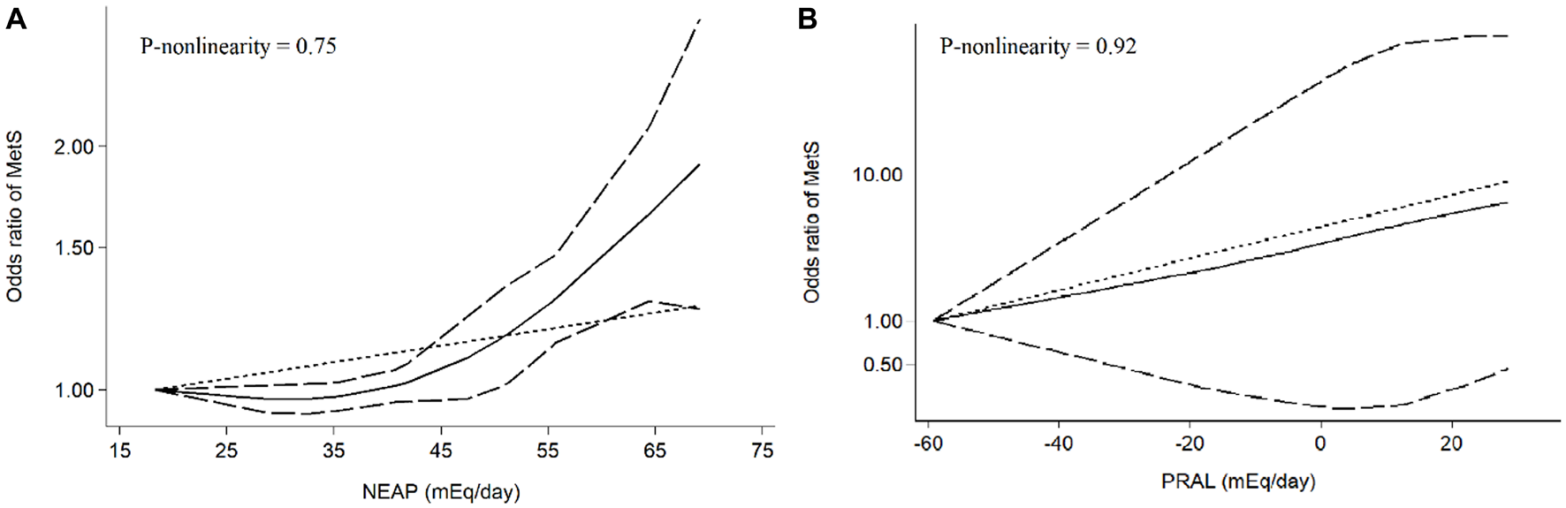
Meta-analysis of the non-linear association between dietary acid load [**(A)** NEAP, **(B)** PRAL] and odds of metabolic syndrome.

**Figure 6:**
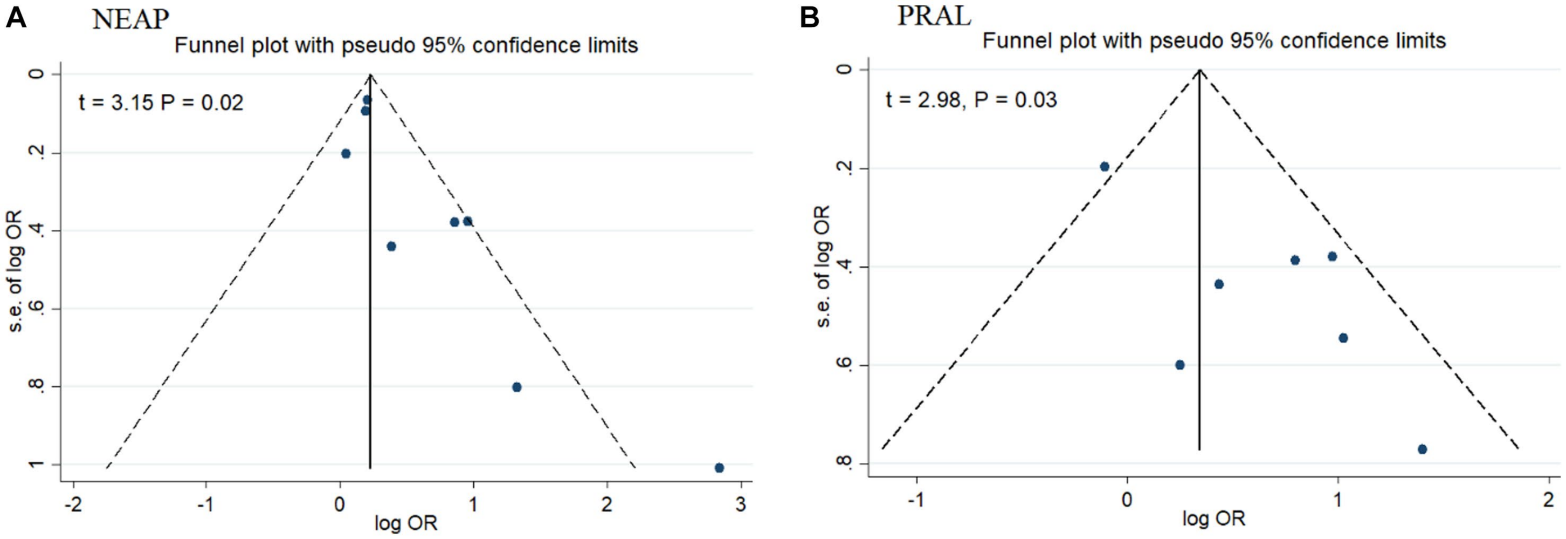
Funnel plot for publication bias in studies on NEAP **(A)** and PRAL **(B)**.

Among seven studies investigating the relation of NEAP to the odds of MetS, four studies (8, 11, 13, 28) identified a significant direct association between diets with high NEAP and odds of MetS. On the other hand, two studies (11, 13) out of seven studies on PRAL found a positive significant relationship between high PRAL and MetS. In the pooled analysis of available evidence by the random-effects model, a significant association between MetS and the indices of the dietary acid load was detected [NEAP: OR = 1.42, 95%CI = 1.12–1.79 (Figure 2); PRAL: OR = 1.76, 95%CI = 1.11–2.78 (Figure 3)]. There was significant evidence of heterogeneity across studies (NEAP: *I*^2^ = 56.4%, *p* = 0.02; PRAL: *I*^2^ = 52.4%, *p* = 0.05). In the linear dose–response meta-analysis, each 10 mEq/day increment in NEAP and PRAL was linked to a 2% (OR = 1.02, 95%CI = 1.001–1.05) and 28% (OR = 1.28, 95%CI = 1.11–1.47) increased odds of MetS, respectively (Figure 4). No evidence for a non-linear relationship was observed for NEAP (P-non-linearity = 0.75) and PRAL (P-non-linearity = 0.92) (Figure 5). The funnel plots revealed a remarkable asymmetry, with significant publication bias in studies on NEAP (Egger’s test *p* = 0.02) and PRAL (Egger’s test *p* = 0.03) (Figure 6).

#### 3.2.1. Subgroup analysis

*In the* subgroup analysis by definition of MetS (Supplementary Figure S1) and the sex of participants (Supplementary Figure S2), the association of NEAP with MetS was supported by studies with NCEP ATPII definition for MetS (OR = 2.53, 95%CI = 1.003–6.43) and studies on both genders (OR = 1.65, 95%CI = 1.01–2.70), but not in men and women subgroups, as well as in studies with JIS and IDF criteria for MetS. Moreover, PRAL was significantly linked to the odds of MetS based on the JIS definition for MetS (OR = 2.22, 95%CI = 1.01–4.78) (Supplementary Figure S3) and in women (OR = 3.17, 95%CI = 1.31–7.65; Supplementary Figure S4).

#### 3.2.2. Sensitivity analysis

In the sensitivity analysis, by removing single studies step by step from the main analysis, no individual study significantly affected the pooled effect sizes for the relation of NEAP and MetS (Supplementary Figure S5), showing the reliability of the findings; however, the sensitivity analysis remarkably affected the relation of PRAL to MetS (Supplementary Figure S6).

## 4. Discussion

The present meta-analysis aimed to investigate the relation of DAL to the odds of MetS. The results identified that higher DAL measured by PRAL and NEAP is significantly associated with elevated odds of MetS. There was a 28 and 2% elevated odds of MetS for each 10 mEq/day increment in PRAL and NEAP, respectively.

Diet is a main environmental factor influencing the odds of MetS (31). Recently, epidemiological evidence has suggested that DAL may play a role in MetS (28). Nevertheless, the available evidence in this regard is contradictory. Iwase et al. identified that, in Japanese patients with type 2 diabetes, higher scores for NEAP and PRAL were linked to the elevated prevalence of MetS (11). In contrast, four cross-sectional investigations on the Iranian population found no relationship between DAL and MetS (9, 12, 29, 30). In agreement with our findings, the study by Arisawa et al. on 14,042 men and 14,105 women revealed that metabolic acidosis, defined by high NEAP, is significantly related to increased odds of MetS (8). Recent meta-analyzes have also demonstrated a close direct relationship between DAL and single components of MetS, including increased obesity (32), impaired glucose metabolism (10), hypertension (33), and dyslipidemia (32), supporting our findings that a diet with a higher DAL may impair metabolic health. The diverging findings of previous studies might result from differences in gender, sociodemographic characteristics of the populations, and the sample size of the studies. Moreover, the varying results may be due to the high differences in the range of PRAL and NEAP across the available evidence. In the present meta-analysis, the strength of association between PRAL with MetS was stronger than NEAP. It has been recognized that PRAL is a more precise indicator of DAL since, in contrast to the NEAP score, which only takes dietary intake of protein and potassium into account, the PRAL score considers dietary intake of protein and several micronutrients, phosphorus, potassium, magnesium, and calcium, as well as the rate at which the nutrients are absorbed in the intestinal border (34). As a result, PRAL is a better predictor of the impacts of diet acidity on health outcomes (10, 35).

Regarding underlying biological mechanisms, high DAL values may be linked to the development of MetS through several interlinked mechanisms, including chronic low-grade inflammation (36), mineral imbalances (37), alterations of the gut microbiota (38), and insulin resistance (39). Chronic low-grade inflammation is a critical component of the pathogenesis of MetS (40). Acidic diets have been reported to induce inflammation by increasing the production of proinflammatory cytokines such as interleukin-6 (IL-6) and tumor necrosis factor-alpha (TNF-α), activating the toll-like receptor (TLR) signaling pathway, and promoting the infiltration of immune cells into adipose tissue (28, 41).The inflammation may further aggravate MetS components such as insulin resistance, hypertension, and dyslipidemia (42). High DAL has been shown to induce insulin resistance, a central process contributing to MetS, by impairing insulin signaling pathways and promoting inflammation and oxidative stress (43). Furthermore, in response to acidic diets, the body releases cortisol (44), a stress hormone that inhibits insulin action, induces lipase activity, and promotes gluconeogenesis, leading to hyperglycemia, hyperinsulinemia, and hypertriglyceridemia (9, 45). The production of acids in the body increases hydrogen ion concentration, leading to a decrease in pH. To neutralize this acidity, the body utilizes alkaline reserves such as bicarbonate, calcium, and magnesium (29, 37). Acidic diets, which are high in animal protein and low in fruits and vegetables (46), tend to decrease the pH of the blood and urine, causing the depletion of alkaline reserves. Such mineral imbalances impair insulin action, disrupt lipid metabolism, and elevate blood pressure, all of which are components of MetS (8, 47). Lastly, a diet with high acidity may lead to dysbiosis of the gut microbiota (38), which has been linked to inflammation, insulin resistance, and dyslipidemia as components of MetS (38, 48). Another mechanism that leads to MetS in response to higher DAL is mediated by increasing adiposity (49), a fundamental component involved in the pathogenesis of all MetS components (9). Accordingly, reducing the intake of acid-forming dietary factors such as animal-based foods, which are high in Western diets, and increasing the consumption of alkaline-forming food items such as fruit, vegetable, potassium, calcium, and magnesium may help prevent or manage MetS (28, 50). Further research is needed to elucidate the mechanisms underlying the association between DAL and MetS and to develop appropriate interventions to mitigate the odds of this disease.

To the best of our knowledge, this was the first meta-analysis evaluating the relation of DAL to MetS. Both NEAP and PRAL scores, as indicators of DAL, were used to analyze the associations with the outcome. We also performed linear and non-linear dose–response analyzes to better understand the pattern of the relationship between DAL and MetS. Despite these strengths, some limitations of our study should be declared. First, all the included publications were cross-sectional in design, and causal inference could not be obtained from the results. While the analyzed data were obtained from large population-based publications with satisfactory quality, prospective and clinical trial studies are required to confirm our findings. Second, there was a remarkable heterogeneity across the studies. This heterogeneity may result from differences in the dietary assessment tools, criteria used to define MetS, genetic background, the level of adjustment for covariates, study population characteristics, and differences in FFQ items in the various populations. Third, significant evidence of publication bias was observed for studies on NEAP and PRAL; our search was limited to English-language publications, which may mean that some studies were ignored. Fourth, even though the majority of the studies controlled the results for potential confounders, residual and unknown confounding factors still might have influenced the findings. Fifth, the included studies were from limited geographic regions (Iran, Japan, and Italy); therefore, the pooled results might not be expandable to all populations. Sixth, calculations of DAL indices were based on self-reported retrospective questionnaires, which are at risk of recall bias. Moreover, the results of the subgroup analyzes should be interpreted with caution because of the small number of the included studies in each subgroup. Sensitivity analysis also revealed that the relation of PRAL to MetS was remarkably affected by single studies, reducing the stability of the results. However, the results were stable for NEAP in the sensitivity analysis. Lastly, DAL is more reliable when diets supply the recommended dietary allowance of protein (0.8 g/kg body weight). In the case of extremely low intake of protein, PRAL would take negative scores; in such a condition, negative scores for PRAL are not representative of an alkaline situation but indicate an unhealthy condition. The included studies did not consider the sufficiency of dietary protein intake and thus may be at risk of an inaccurate estimation of diet-related acidosis.

## 5. Conclusion

The results of the present meta-analysis propose that high DAL is related to the increased prevalence of MetS. Additional studies, particularly prospective cohort and clinical trials, are needed to elucidate the association between DAL and MetS and to reveal the underlying mechanisms.

## Data availability statement

The original contributions presented in the study are included in the article/Supplementary material, further inquiries can be directed to the corresponding author.

## Author contributions

SA-H, FM, SSA, NG, and RA: conceptualization and software. ES, NG, SNA, KO, and RR-P: methodology. BH, NG, FM, SNA, and SSA: validation. NG, BH, FM, and RR-P: formal analysis. SA-H, RA, ES, and KO: investigation. SA-H, FM, SSA, RA, ES, and KO: data curation and writing–original draft preparation. SNA, NG, BH, and RR-P: writing–review and editing. SA-H: visualization and project administration. SA-H and NG: supervision and funding acquisition. All authors contributed to the article and approved the submitted version.

## Conflict of interest

The authors declare that the research was conducted in the absence of any commercial or financial relationships that could be construed as a potential conflict of interest.

## Publisher’s note

All claims expressed in this article are solely those of the authors and do not necessarily represent those of their affiliated organizations, or those of the publisher, the editors and the reviewers. Any product that may be evaluated in this article, or claim that may be made by its manufacturer, is not guaranteed or endorsed by the publisher.
