# Supplementary material for: Metabolic syndrome in relation to dietary acid load: a dose–response meta-analysis of observational studies
Source: Front Nutr. 2023 Aug 11;10:1233746. doi: 10.3389/fnut.2023.1233746 (PMC10450920; doi:10.3389/fnut.2023.1233746)
Supplement: Supplementary file 1 [file Data_Sheet_1.docx]

**Online Supporting Material**

Supplemental Table 1. Quality assessment of studies included in this systematic review and meta-analysis based on the Newcastle-Ottawa Scale (NOS) criteria

|  | Representativeness of the sample | Sample size | Non-respondents | Ascertainment of exposure | Study controls for energy intake | Study controls for any additional factor | Ascertainment of outcome | Statistical test | **Total score** |
| --- | --- | --- | --- | --- | --- | --- | --- | --- | --- |
| Arisawa et al. 2020 | * | * | * | * | * | * | ** | * | 9 |
| Sanz et al. 2022 | * | * | * | * | NA | NA | ** | * | 7 |
| Iwase et al. 2015 | * | NA | * | * | * | * | ** | * | 8 |
| Jafari et al. 2021 | * | * | NA | * | * | * | ** | * | 8 |
| Mozaffari et al. 2019 | * | * | NA | * | * | * | ** | * | 8 |
| Rezazadegan et al. 2022 | * | * | NA | * | * | * | ** | * | 8 |
| Tangestani et al. 2022 | * | * | NA | * | NA | * | ** | * | 7 |
| Mohammadifard et al. 2020 | * | * | * | * | NA | * | ** | * | 8 |

NA: Not Applicable


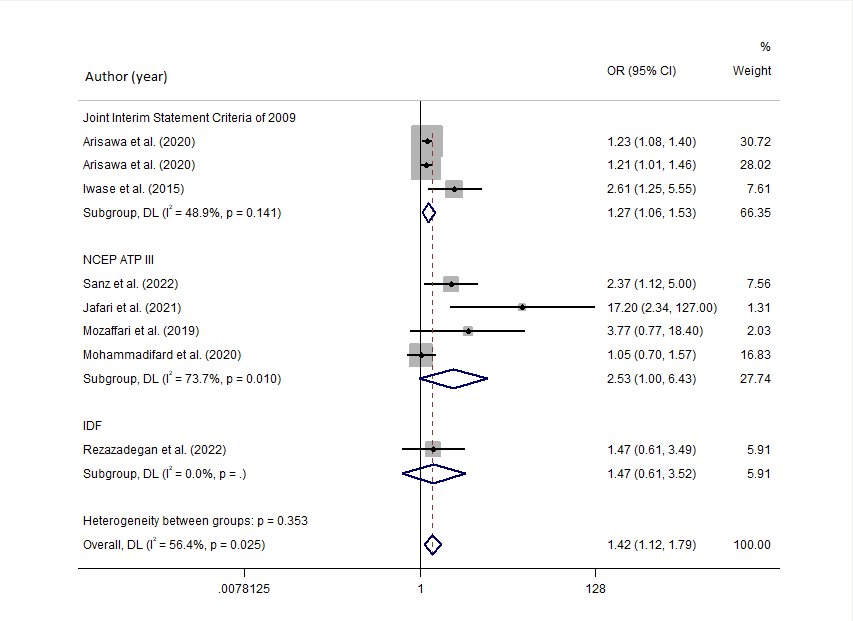


Supplemental figure 1. Subgroup analysis for the association of NEAP with the odds of MetS according to the definition of MetS


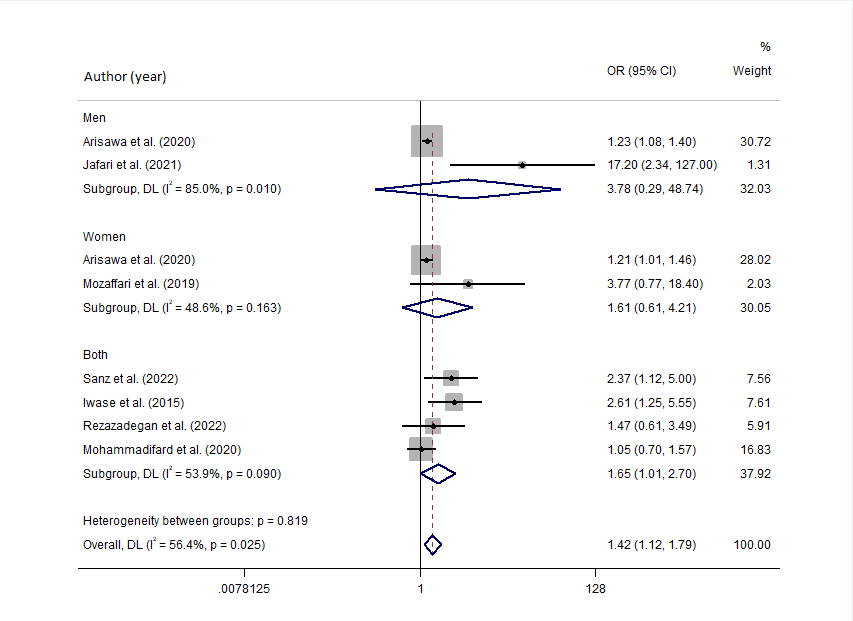


Supplemental figure 2. Subgroup analysis for the association of NEAP with the odds of MetS according to the sex of participant


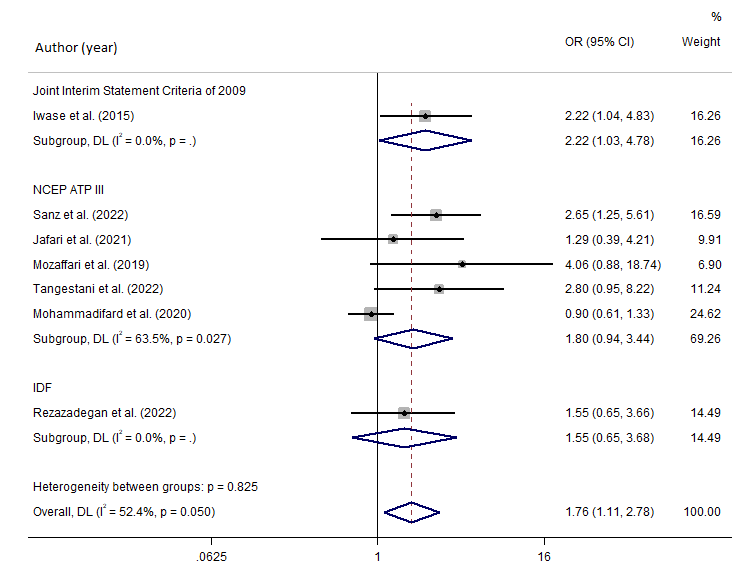


Supplemental figure 3. Subgroup analysis for the association of PRAL with the odds of MetS according to the definition of MetS


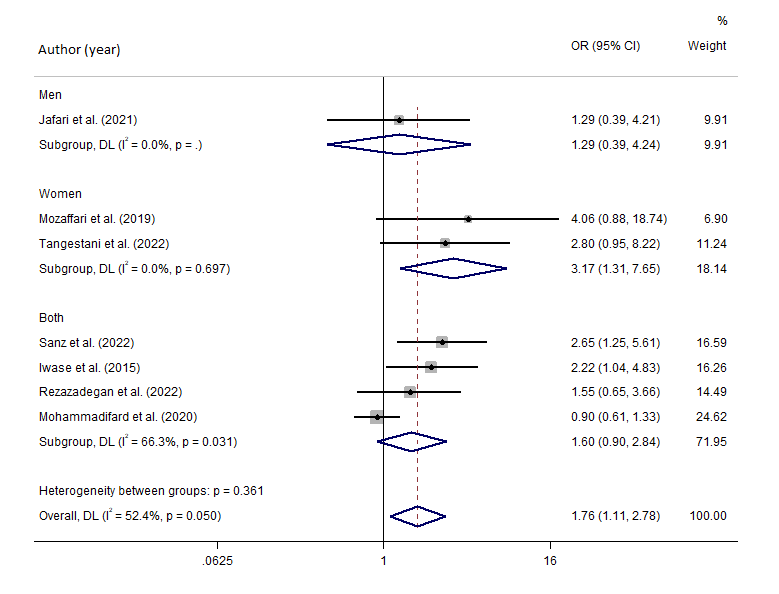


Supplemental figure 4. Subgroup analysis for the association of PRAL with the odds of MetS according to the sex of participants

Supplemental figure 5. Sensitivity analysis for the association of NEAP with the odds of MetS

Supplemental figure 6. Sensitivity analysis for the association of PRAL with the odds of MetS
